# A novel six-microRNA-based model to improve prognosis prediction of breast cancer

**DOI:** 10.18632/aging.101767

**Published:** 2019-01-30

**Authors:** Jianguo Lai, Hongli Wang, Zihao Pan, Fengxi Su

**Affiliations:** ^1^ Guangdong Provincial Key Laboratory of Malignant Tumor Epigenetics and Gene Regulation, Sun Yat-Sen Memorial Hospital, Sun Yat-Sen University, Guangzhou, China; ^2^ Breast Tumor Center, Sun Yat-Sen Memorial Hospital, Sun Yat-Sen University, Guangzhou, China; ^3^ Department of Thoracic Surgery, Sun Yat-Sen Memorial Hospital, Sun Yat-Sen University, Guangzhou, China

**Keywords:** breast cancer, microRNA, nomogram, survival, model

## Abstract

Current tumor-node-metastasis (TNM) stage is unable to accurately predict the overall survival (OS) in breast cancer (BC) patients. This study aimed to construct a microRNA (miRNA)-based model to improve survival prediction of BC. We confirmed 99 differentially expressed miRNAs (DEMs) in 1044 BC samples compared to 102 adjacent normal breast tissues from The Cancer Genome Atlas (TCGA) database. Prognostic DEMs were used to establish a miRNA-based nomogram via Cox regression model. Gene ontology (GO) and Kyoto Encyclopedia of Genes and Genomes analyses (KEGG) were executed to analyze target genes of miRNAs. A six-miRNA signature was screened to effectively distinguish high-risk patients in the primary and validation cohort (all *P*<0.001). Furthermore, we established a novel prognostic model incorporating the six-miRNA signature and clinical risk factors to predict 5-year OS of BC. Time-dependent receiver operating characteristic analysis suggested that the predictive accuracy of the six-miRNA-based nomogram was distinctly higher than that of TNM stage (0.758 *vs* 0.650, *P*<0.001). GO and KEGG pathway analyses showed that the 39 target genes mainly enrichment in protein binding, cytoplasm and MAPK signaling pathway. Our six-miRNA-based model is a reliable prognostic tool for survival prediction and provides information for individualized treatment decisions in BC patients.

## INTRODUCTION

Breast cancer (BC) is an enormous public health burden worldwide and ranks as the main cause of cancer deaths in China [1]. With the advances of the comprehensive therapeutic strategies, the 5-year overall survival (OS) rate of BC has been improve dramatically. However, BC kills about 1.2 million people in China each year [2]. Prognostic evaluation is vital for making appropriate therapeutic decisions and follow-up strategies in BC patients. Currently, tumor-node-metastasis (TNM) stage is a key tool for prognostic assessment and a specific treatment choices. However, BC patients at same TNM staging can have very different clinical outcomes. The traditional TNM staging system is mainly on the basis of anatomical information, which is unable to sufficient prediction for prognosis of individual patients because it could not display the biological heterogeneity of BC [3]. Therefore, a predictive tool that can integrate molecular biomarkers into the TNM staging system may improve the accuracy of survival prediction for BC patients.

MicroRNAs (miRNAs) are small, non-coding single-stranded RNAs (18–25 nucleotides) and negatively regulate gene expression by base-pair matching with the 3′UTRs of target mRNAs [4]. Accumulating evidence shows that miRNAs play critical roles in various physiological and pathological processes, including metabolism, carcinogenesis, and proliferation [4, 5]. In addition, previous reports have suggested the important prognostic value of miRNAs signature in a variety of cancers [6–20]. But most of these studies were based on limited number of patients and different miRNAs platforms, lack a normalized standard. Thus, the Cancer Genome Atlas (TCGA) database provides us with a comprehensive catalogue of large-scale miRNAs expression data. Besides, the prognostic value of miRNAs signature to predict 5-year OS of BC patients has not been fully illustrated. With the ability of incorporating diverse independent prognostic variables to provide an individual probability of survival outcome, nomogram is widely applied for cancer prognosis [3, 21].

Therefore, this study aimed to construct a novel miRNA-based model to improve survival prediction and effectively pick out the high-risk patients based on TCGA miRNA sequencing data. Such a practical tool has the potential to guide more effective individualized treatment decisions for BC patients.

## RESULTS

### Baseline characteristics of patients

A total of 984 BC patients from TCGA database were included. The detailed baseline characteristics of the primary and validation cohort were listed in Table 1. No significant difference of baseline characteristics were displayed between the two independent cohort in Table 1 (all P>0.05). The median age of the 984 BC patients was 58 year (interquartile range [IQR]: 48–67). The 5-year OS rate of the 984 BC patients was 82.6%.

**Table 1:** Baseline characteristics of study patients

Variables	Primary cohort No. (%)	Validation cohort No. (%)	P-value
**No. of patients**	984	492	
**Age (years)**	58(48,67)	58(48,67)	0.767
**T stage**			0.620
T1	262(26.6)	132(26.8)	
T2	570(57.9)	295(59.9)	
T3	123(12.5)	50(10.2)	
T4	29(3.0)	15(3.1)	
**N stage**			0.987
N0	453(46.0)	222(45.1)	
N1	341(34.7)	169(34.4)	
N2	107(10.9)	56(11.4)	
N3	72(7.3)	39(7.9)	
Unknown	11(1.1)	6(1.2)	
**TNM stage**			0.980
I	170(17.3)	82(16.7)	
II	569(57.8)	284(57.7)	
III	226(23.0)	117(23.8)	
IV	19(1.9)	9(1.8)	
**ER status**			0.925
Negative	206(20.9)	101(20.5)	
Positive	738(75.0)	369(75.0)	
Unknown	40(4.1)	22(4.5)	
**PR status**			0.878
Negative	262(26.6)	137(27.9)	
Positive	598(60.8)	295(59.9)	
Unknown	124(12.6)	60(12.2)	
**HER2 status**			0.980
Negative	679(69.0)	337(68.5)	
Positive	152(15.5)	77(15.6)	
Unknown	153(15.5)	78(15.9)	

**Abbreviations:** TNM, tumor-node-metastasis; ER, estrogen receptor; PR, progesterone receptor; HER2, human epithelial growth factor receptor 2.

### Candidate OS-related miRNAs of BC patients in the primary cohort

**Figure 1:**
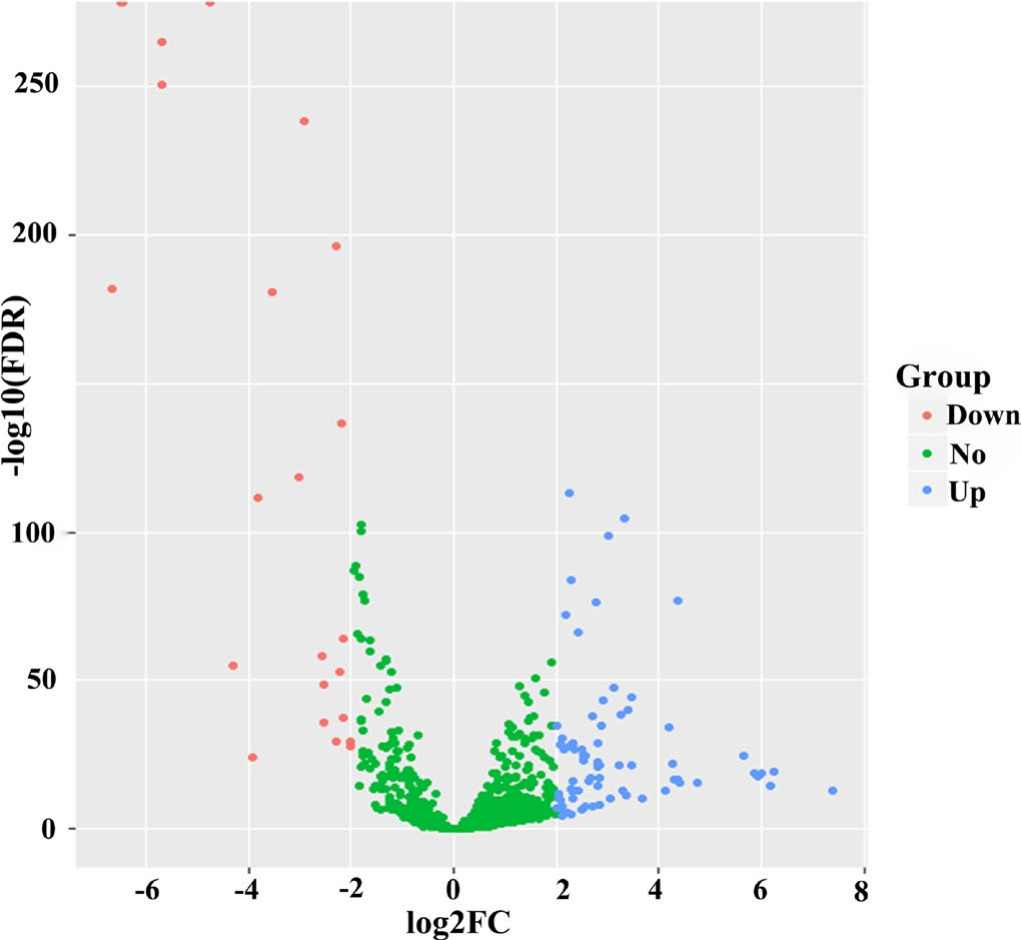
Volcano plot of 1601 miRNAs in breast cancer patients. Blue color indicates up-regulated expression, and red color represents down-regulated expression.

On the basis of the TCGA database, 99 differentially expressed miRNAs (DEMs) (false discovery rate (FDR)<0.05 and |log2fold change (log2FC)|≥2) were identified using 1601 miRNAs expression profiles between 1044 BC samples and 102 adjacent normal breast tissues. The volcano plot of these 1601 miRNAs were visualized via the “ggplot2” package of R software in Figure 1. These 99 DEMs were found as potential prognostic miRNAs for BC patients, among which 75 miRNAs were confirmed as upregulated and 24 as downregulated. To pick out the OS-related miRNAs, 99 DEMs were initially subjected to univariate Cox proportional hazards regression (CPHR) analysis in the primary cohort. Then, 10 miRNAs (hsa-miR-551b, hsa-miR-210, hsa-miR-6715a,hsa-miR-147b, hsa-miR-203b, hsa-miR-4501, hsa-miR-4446, hsa-miR-7974, hsa-miR-4675, hsa-miR-549a) were distinctly associated with OS of BC patients (*P* <0.05) and were subsequently selected into a multivariate CPHR analysis. Finally, six DEMs (five risky miRNAs: hsa-miR-549a, hsa-miR-6715a, hsa-miR-4501, hsa-miR-7974,hsa-miR-4675; one protective miRNA: hsa-miR-147b) were confirmed as independent prognostic miRNAs of BC patients in the primary cohort (Table 2).

**Table 2:** Six prognostic miRNAs significantly associated with OS in the primary cohort

Name	Coefficient	Type	Down/up-regulated	HR	95%CI	P value
**hsa-miR-147b**	-0.054	Protective	Up	0.947	0.902-0.995	0.032
**hsa-miR-549a**	0.289	Risky	Up	1.336	1.140-1.564	<0.001
**hsa-miR-6715a**	0.072	Risky	Down	1.075	1.029-1.122	0.001
**hsa-miR-4501**	0.026	Risky	Up	1.026	1.009-1.044	0.004
**hsa-miR-7974**	0.158	Risky	Up	1.171	1.083-1.266	<0.001
**hsa-miR-4675**	0.068	Risky	Up	1.07	1.012-1.132	0.018

**Abbreviations:** OS, overall survival; CI, confidence interval.

### Development of risk score formula and six-miRNA-based prognostic model

**Figure 2:**
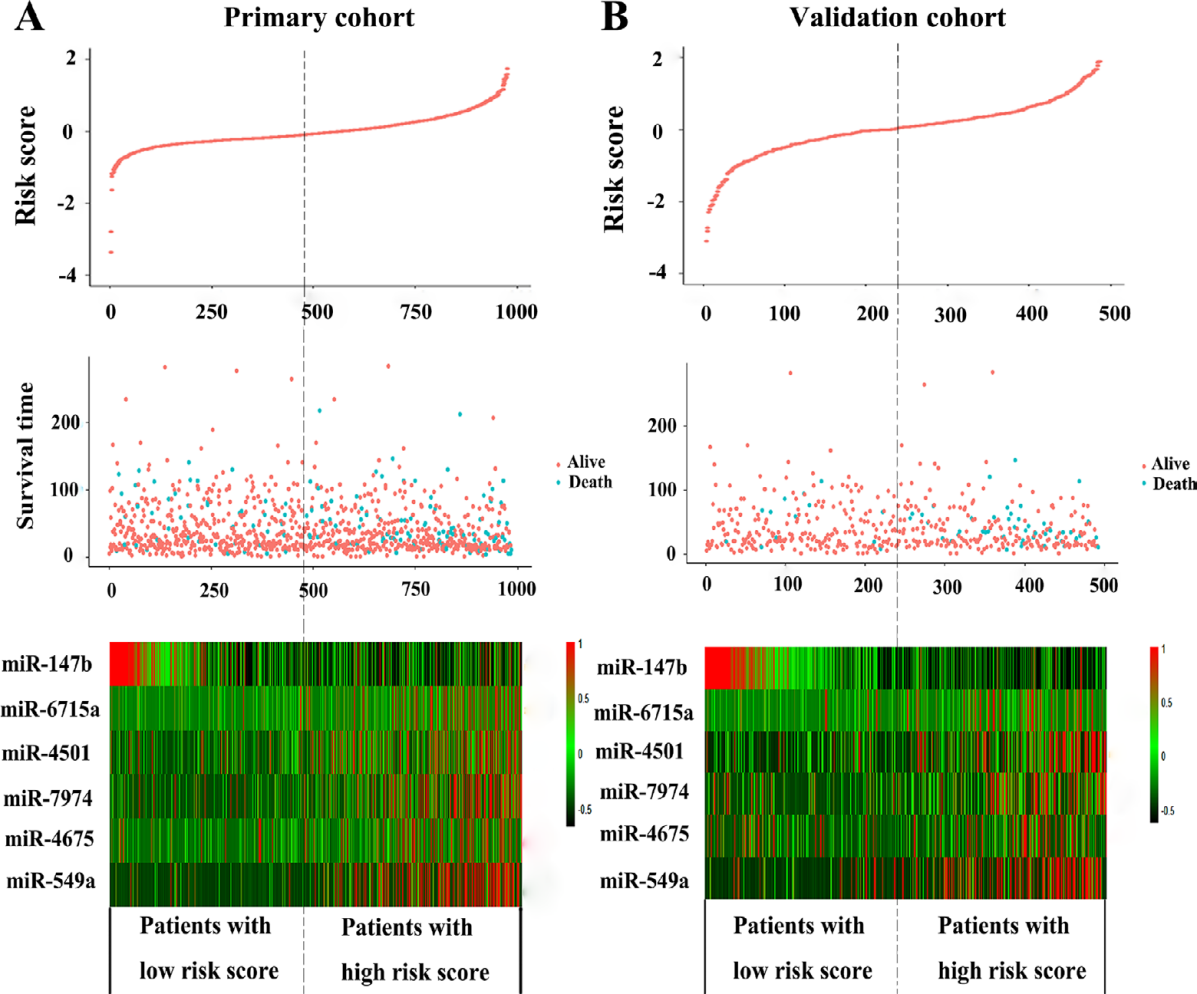
The distribution of risk score, OS, and OS status and the heat map of prognostic six-miRNA signature in the primary cohort (**A**) and validation cohort (**B**). The dotted line indicates the cutoff point of the median risk score used to stratify patients into the low-risk group and high-risk group. OS, overall survival.

**Figure 3:**
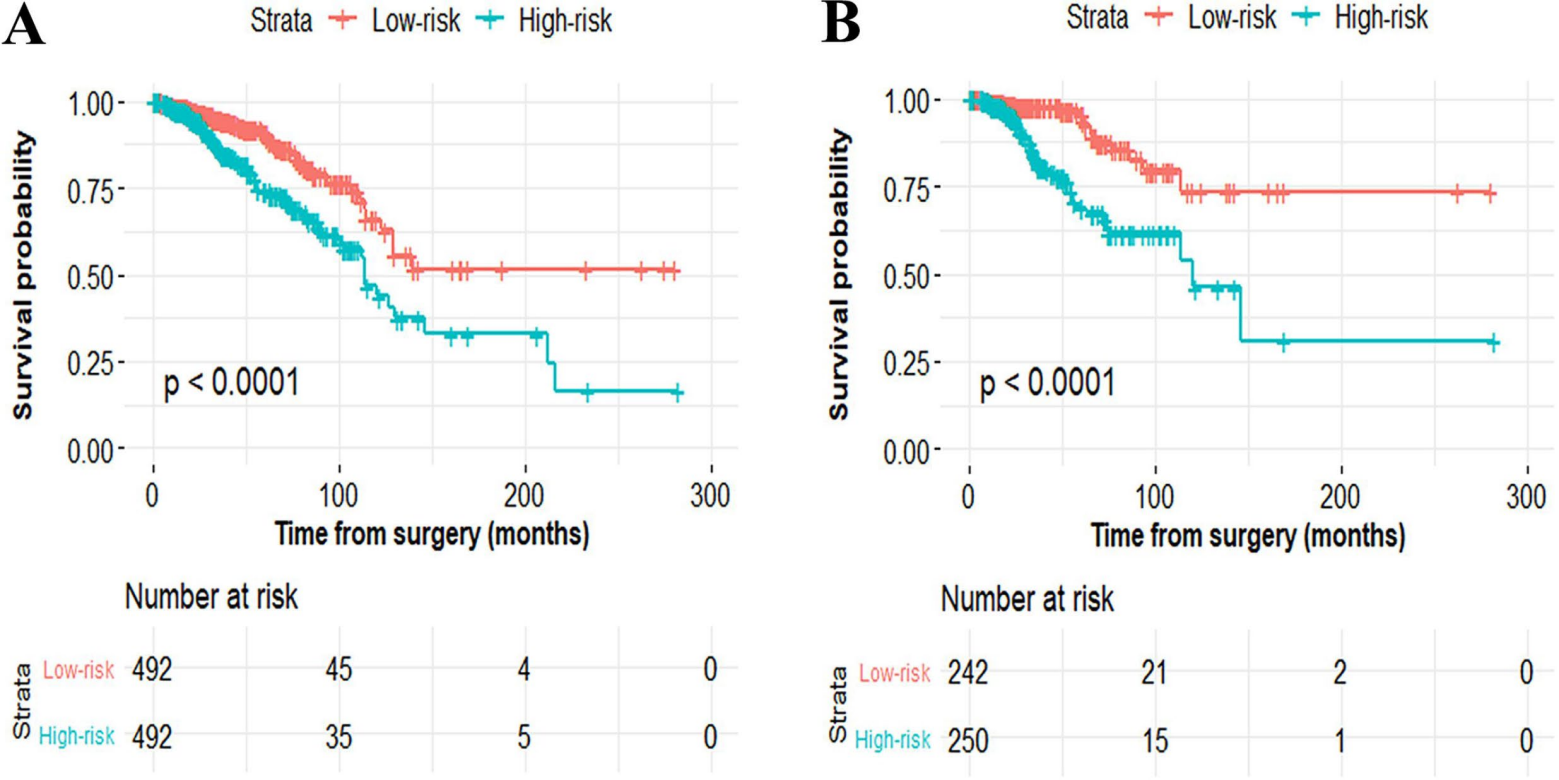
Kaplan–Meier curves of overall survival for breast cancer patients based on the six-miRNA signature in the primary cohort (**A**) and validation cohort (**B**).

To facilitate the utility of the identified prognostic miRNAs in routine clinical practice, the following formula was developed to generate risk score for each patient: Risk score=(0.289×expression_miR-549a_)+ (0.072× expression_miR-6715a_)+(0.026×expression_miR-4501_)+ (0.158× expression_miR-7974_)+(0.068×expression_miR-4675_)-(0.054× expression_miR-147b_). Thus, patients were classified into the low-risk group and the high-risk group via the same median risk score as the cut-off point in the two independent cohorts. The distributions of the miRNA-based risk scores, OS, OS status, and six-miRNA expression profiles of the training cohort and validation cohort are showed in Figure 2. The heat map suggests that the five risky miRNAs (hsa-miR-549a, hsa-miR-6715a, hsa-miR-4501, hsa-miR-7974, hsa-miR-4675) have high expression in the high-risk group, while the one protective miRNA (hsa-miR-147b) exhibits high expression in low-risk group (Figure 2). Besides, compared with the low-risk group, Kaplan–Meier survival analysis shows that the high-risk group has a obvious poorer prognosis (*P*<0.0001) (Figure 3).

**Figure 4:**
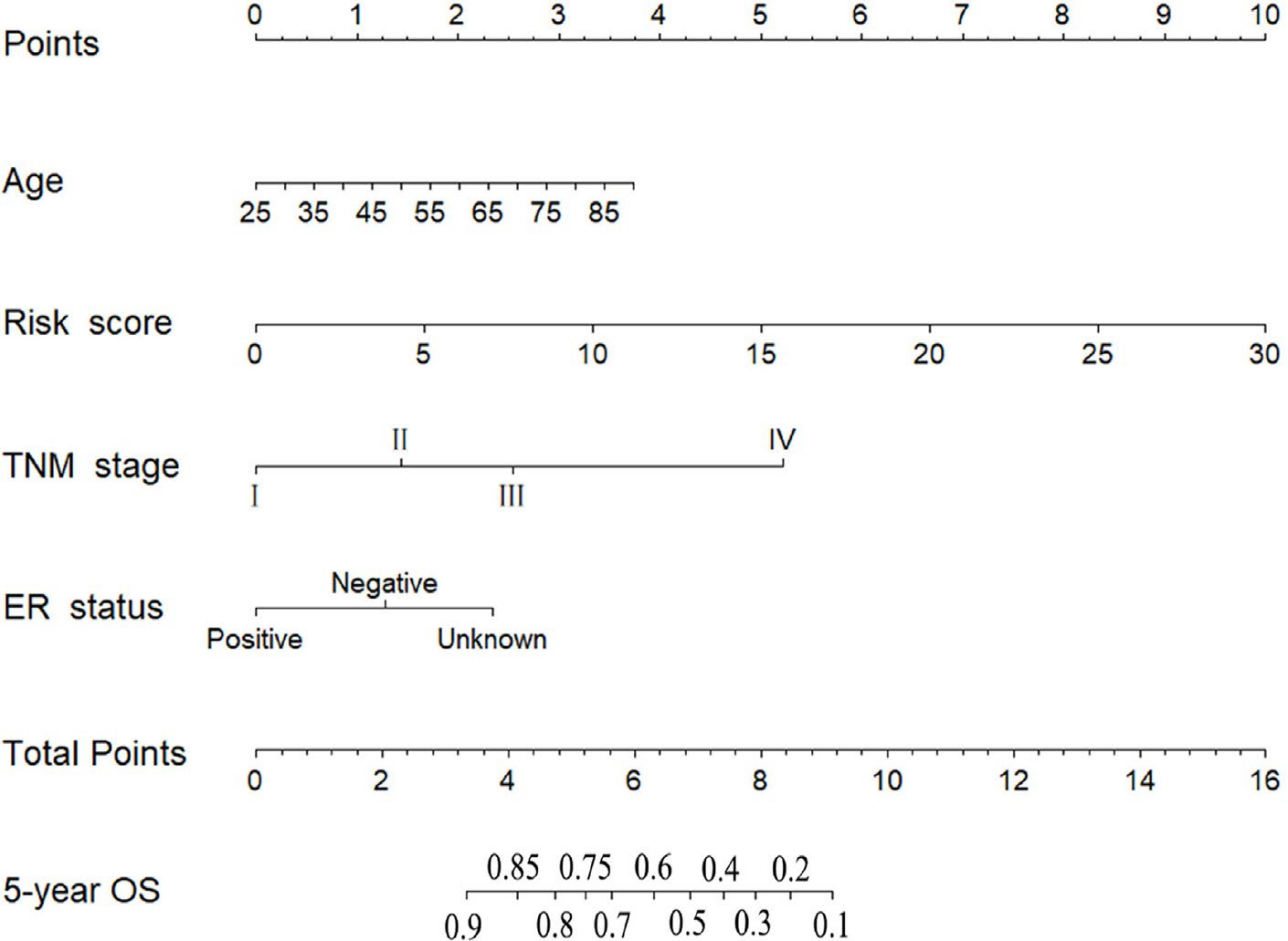
Six-miRNA-based prognostic model to predict 5-year overall survival in breast cancer patients.

According to the results of univariate and multivariate CPHR analyses (Table 3), the six-miRNA signature and three clinical risk factors (age, TNM stage and ER status) were identified as independent prognostic variables of OS. T stage and N stage were not entered into multivariate CPHR analysis, because they were associated with TNM stage, known as multicollinearity, could lead to spurious associations and unreliable results [21]. To construct a more sensitive predictive tool in clinical practice, we built a novel six-miRNA-based prognostic model integrating the six-miRNA signature and three clinical risk factors (age, TNM stage and ER status) to predict 5-year OS of BC patients (Figure 4). The six-miRNA-based nomogram revealed the six-miRNA signature and TNM stage as the largest contribution to 5-year OS, followed by the age and ER status. Each variable was acquired a nomogram score on the point scale. After calculating the total nomogram score, we could easily obtain the nomogram-predicted probability of 5-year OS for each patient.

**Table 3:** Univariate and multivariate Cox proportional hazards regression analyses in the primary cohort

Variables	Univariate analysis		Multivariate analysis	
	Hazard ratios (95%CI)	*P*-value	Hazard ratios (95%CI)	*P*-value
**Age**	1.029(1.016-1.042)	**<0.001**	1.031(1.017-1.045)	**<0.001**
**T stage**				
T1	Referent			
T2	1.620(1.037-2.530)	**0.034**		
T3	1.623(0.911-2.889)	**0.100**		
T4	4.402(2.194-8.830)	**<0.001**		
**N stage**				
N0	Referent			
N1	2.035(1.350-3.069)	**0.001**		
N2	2.977(1.745-5.081)	**<0.001**		
N3	4.246(2.287-7.882)	**<0.001**		
Unknown	7.666(3.001-19.582)	**<0.001**		
**TNM stage**				
I	Referent		Referent	
II	2.011	**0.021**	2.142(1.179-3.893)	**0.012**
III	3.269	**<0.001**	3.850(2.042-7.256)	**<0.001**
IV	12.784	**<0.001**	15.909(7.474-33.865)	**<0.001**
**ER status**				
Negative	Referent		Referent	
Positive	0.652(0.444-0.958)	**0.029**	0.508(0.342-0.754)	**0.001**
Unknown	1.918(0.921-3.997)	0.082	1.755(0.837-3.678)	**0.137**
**PR status**				
Negative	Referent			
Positive	0.737(0.498-1.090)	0.127		
Unknown	1.260(0.757-2.098)	0.374		
**HER2 status**				
Negative	Referent			
Positive	1.288(0.778- 2.134)	0.325		
Unknown	1.459(0.969-2.196)	0.071		
**Six-miRNA signature**	1.194(1.140-1.252)	**<0.001**	1.193(1.138-1.251)	**<0.001**

**Notes:** Bold values indicate statistical significance (P<0.05). CI, confidence interval, ER, estrogen receptor; PR, progestrone receptor; HER2, human epithelial growth factor receptor 2.

### Assessment of the six-miRNA-based signature and prognostic model

**Figure 5:**
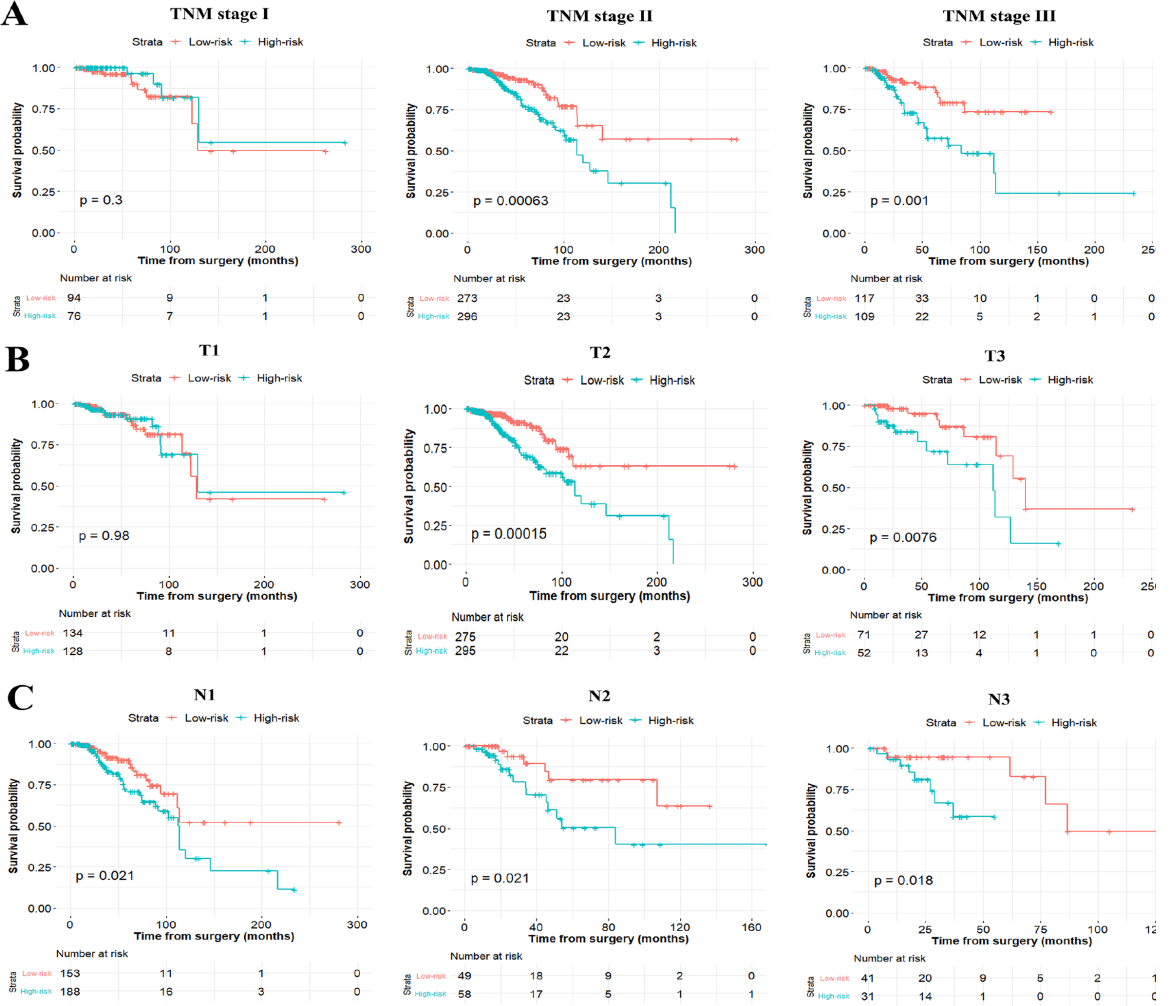
Stratified analysis of the six-miRNA signature for breast cancer patients in TNM stage (**A**), T stage (**B**), and N stage (**C**).

**Figure 6:**
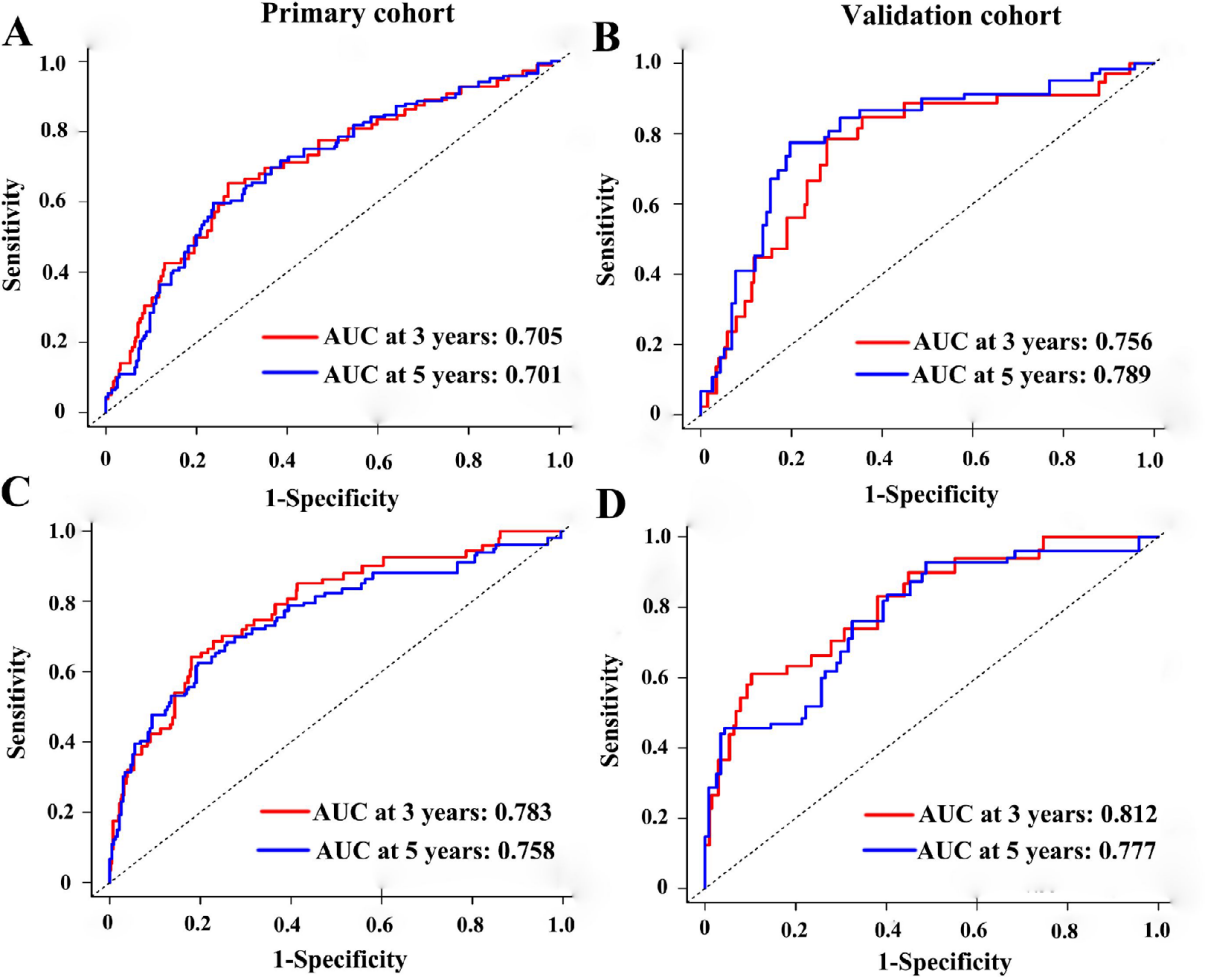
Time-dependent receiver operating characteristic curves at 3-, 5-years based on the six-miRNA signature in the primary cohort (**A**) and validation cohort (**B**). Time-dependent receiver operating characteristic curves at 3-, 5-years based on the six-miRNA-based prognostic model in the primary cohort (**C**) and validation cohort (**D**).

**Figure 7:**
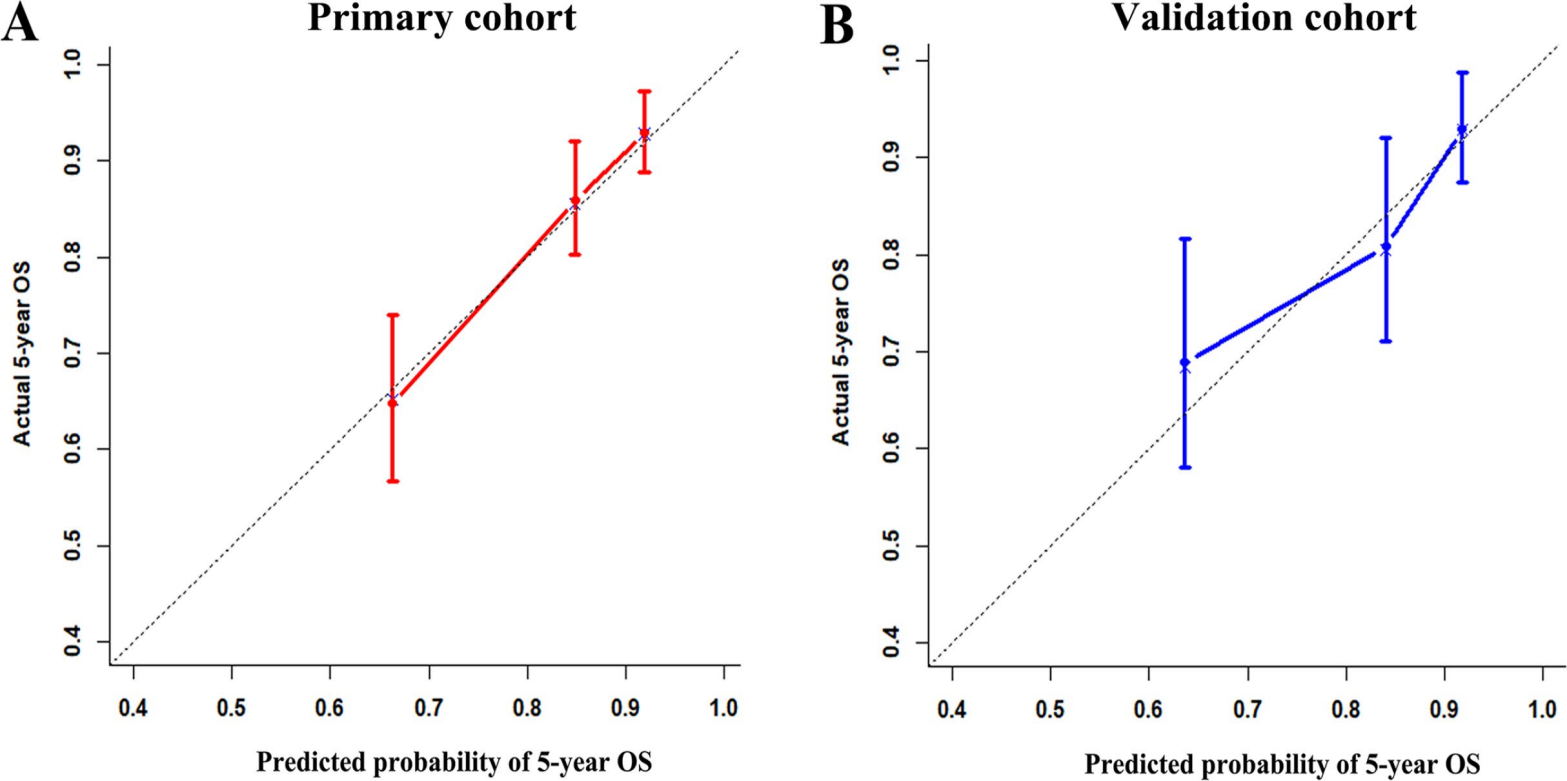
Calibration plots of the six-miRNA-based prognostic model in the primary cohort (**A**) and validation cohort (**B**).

To test whether the six-miRNA signature could predict OS regardless of stages, we performed risk stratification in patients with TNM stage, T stage, and N stage. The patients with low-risk scores had significantly better OS than patients with high-risk scores in TNM stage II (*P*=0.00063), TNM stage III (*P*=0.001), T2 (*P*=0.00015), T3 (*P*=0.0076), N1 (*P*=0.021), N2 (*P*=0.021) and N3 (*P*=0.018) (Figure 5). To assess the predictive performance of the six-miRNA-based signature and prognostic nomogram, we conducted a time-dependent ROC curve analysis by comparing the respective AUC value. Then, the AUC values of the six-miRNA signature at 5 years were 0.701 (95%CI: 0.633–0.768) and 0.789 (95%CI: 0.715–0.880) in the primary cohort and validation cohort, respectively (Figure 6A–6B). And the AUC values of the six-miRNA-based prognostic model at 5 years were 0.758 (95%CI: 0.686–0.830) and 0.777 (95%CI: 0.687–0.867) in the primary cohort and validation cohort, respectively (Figure 6C–6D). Importantly, these AUC values revealed that six-miRNA-based signature and prognostic nomogram had favorable discrimination performance for BC patients. In addition, calibration plots of the six-miRNA-based prognostic model fitted well in the training cohort and validation cohort, which indicated good calibration ability (Figure 7).

### Comparison with other prognostic factors

**Figure 8:**
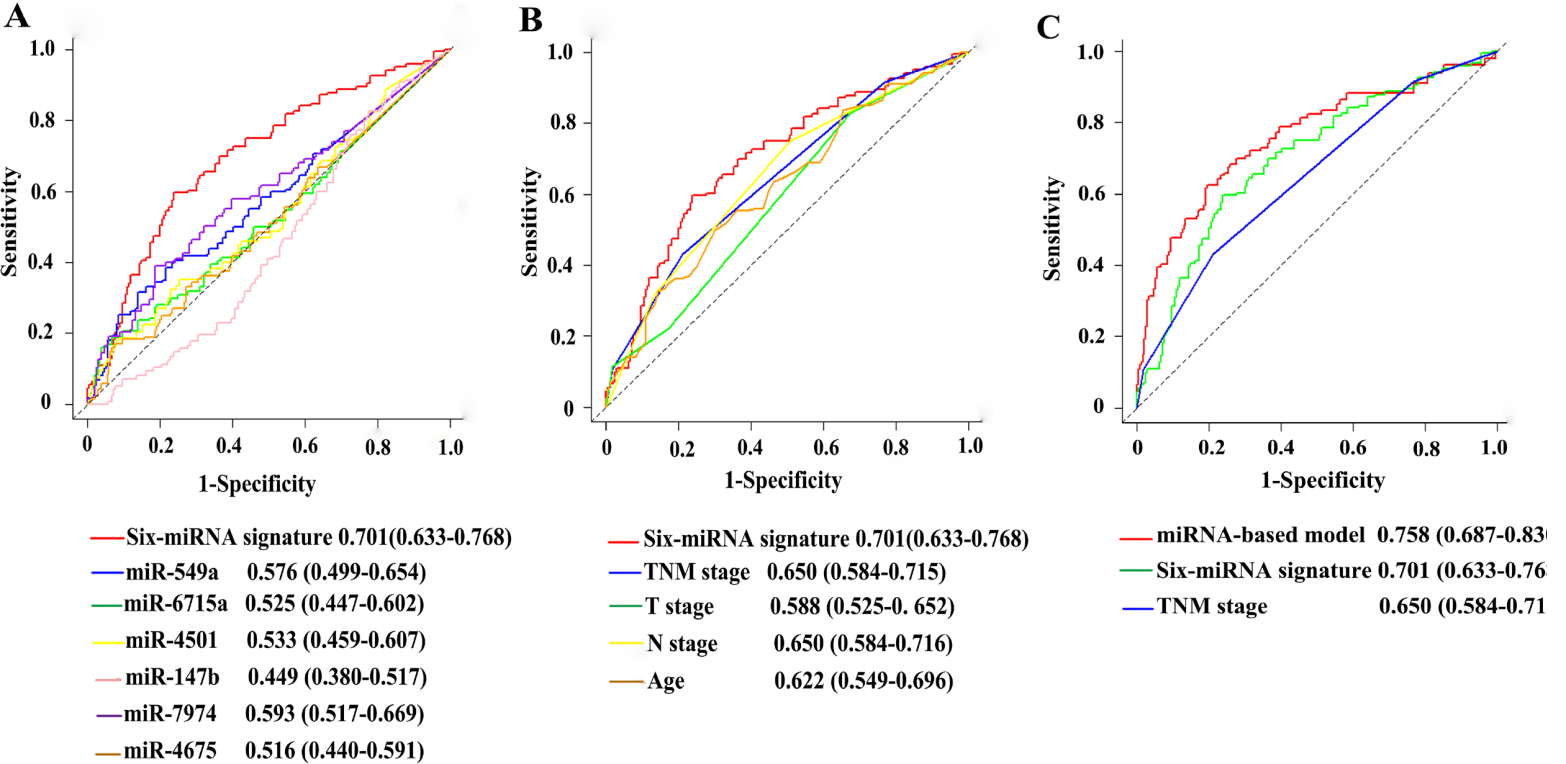
Comparisons of the prognostic accuracy at 5-years using time-dependent receiver operating characteristic curves in the six-miRNA signature with single miRNA (**A**), the six-miRNA signature with clinical risk factors (**B**), and the six-miRNA-based prognostic model with six-miRNA signature, TNM stage (**C**).

In ROC analysis to compare predictive accuracy of different prognostic factors, the six-miRNA signature suggested higher prognostic accuracy than clinical risk factors, or single miRNA alone (Figure 8A–8B). Thus, the six-miRNA signature can outperform the clinical prognostic features. More importantly, the six-miRNA-based prognostic nomogram had significantly better predictive performance than TNM stage (0.758 *vs* 0.650, *P*<0.001) (Figure 8C).

### GO and KEGG pathway analyses of predicted target genes

**Figure 9:**
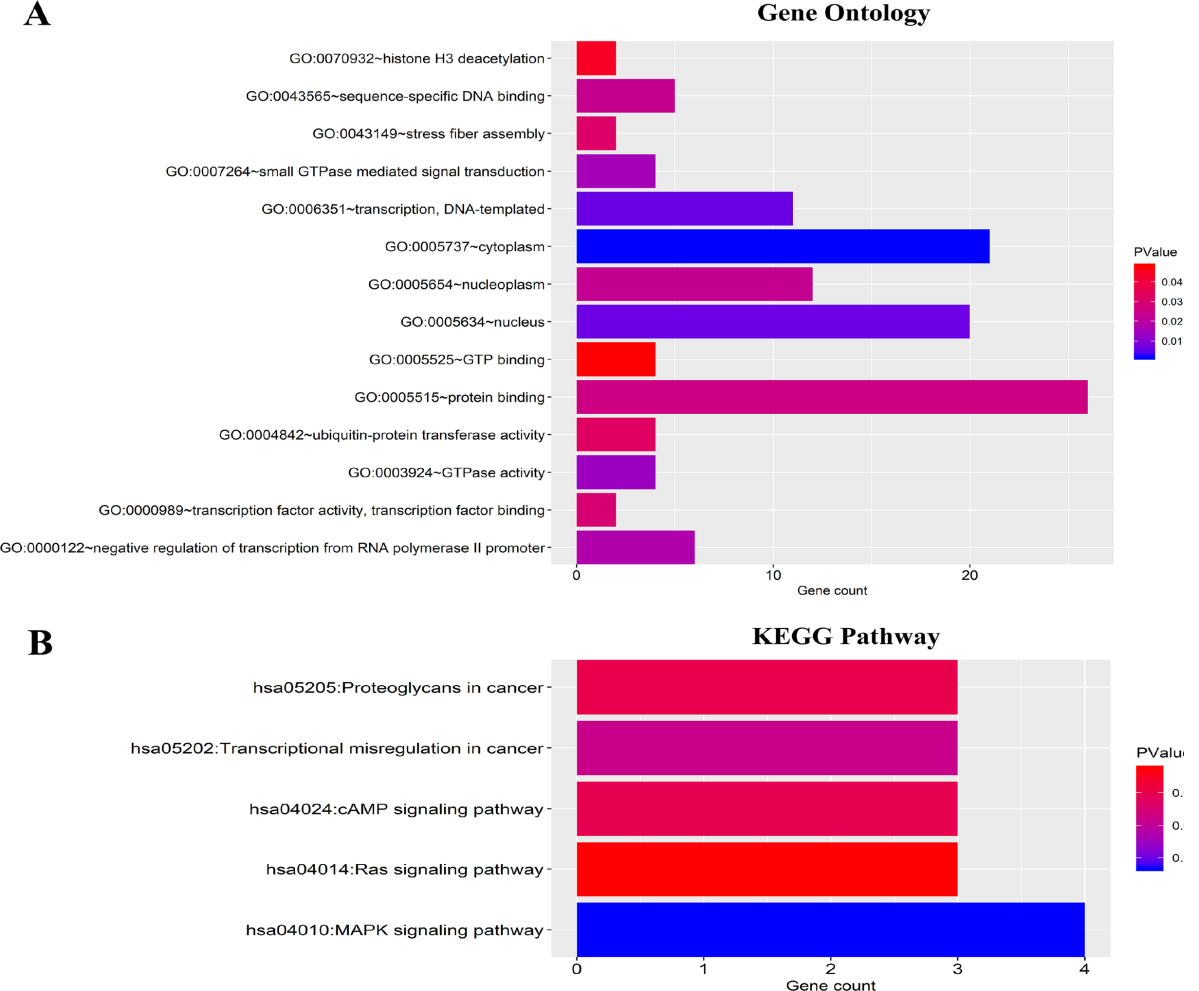
Functional enrichment analysis for predicted target genes of the six miRNAs. (**A**) Gene ontology (GO) enrichment analysis. (**B**) Kyoto Encyclopedia of Genes and Genomes analyses (KEGG) enrichment analysis. The x-axis indicates the number of genes, and the y-axis represents the GO terms and KEGG pathway names. The color represents the P-value.

To evaluate the potential function of the six-miRNAs, a total of 39 target genes of the six-miRNAs were predicted using TargetScan, miRTarBase and miRDB database, respectively. GO analysis included molecular function (MF), biological process (BP), and cellular component (CC). The 39 target genes were mainly related with protein binding (MF), transcription and DNA-templated (BP), cytoplasm and nucleus (CC) (Figure 9A). And KEGG pathway analysis revealed that the 39 genes mainly enriched in MAPK signaling pathway, transcriptional misregulation in cancer and cAMP signaling pathway (Figure 9B).

## DISCUSSION

A molecular marker-based approach to accurately predict survival in BC patients is urgently needed in the era of precision medicine. Accumulating evidence indicates that miRNAs play a vital role in BC prognosis[10, 22–24]. In the present study, we confirmed six-miRNA signature that was significantly associated with OS in BC patients based on the TCGA database. Furthermore, this six-miRNA signature enabled to stratify patients into the low-risk and high-risk groups with distinct differences in 5-year OS. Moreover, a novel six-miRNA-based prognostic model combining six-miRNA signature and clinical risk factors was established and validated to improve survival prediction for BC patients. The six-miRNA-based nomogram consisted of four independent prognostic variables, including age, TNM stage, ER status, and six-miRNA signature. The proposed tool was significantly superior to the traditional TNM stage in predicting 5-year OS for BC patients. The AUC value of the six-miRNA-based prognostic model was 0.758, which indicating favorable discrimination performance. Therefore, our six-miRNA-based nomogram might be a vital tool for survival prediction in BC patients, aiding in personalized therapeutic treatment strategies and postoperative counseling. Further bioinformatics analysis helps us understand the biological function of the six OS-related miRNAs. On the basis of the GO and KEGG pathway analyses, the six-miRNAs may play crucial roles in protein binding, transcription and DNA-templated, cytoplasm, nucleus, MAPK signaling pathway, transcriptional misregulation in cancer and cAMP signaling pathway.

Previous reports about DEMs have indicated that the miRNA-based signature is a important marker for survival or relapse in a variety of cancers [8–10, 12, 14, 23–27]. Recently, Gong et al built a miRNA-based classifier to predict relapse in Hormone Receptor-Positive HER2-Negative BC patients [24]. However, this study has been limited by small sample size and small number of miRNAs screened to mine miRNA expression profiling. In addition, many researches were inconsistent in these sets of prognostic miRNAs because of the heterogeneous of BC and variations in the methods for miRNAs selection. TCGA database provides a robust platform to systematically analyze the large-scale miRNA sequencing data. Consequently, compared with above previous study, a total of 1601 miRNAs were initially selected in our study, which could provide a more comprehensive analysis. Besides, the miRNA signature from the TCGA database regarding to the 5-year OS of BC patients has not been reported.

Although the miRNA-based model performs well in BC survival prediction, there are several shortcomings should be acknowledged in the present study. First, experimental studies should be conducted to deeply explore the molecular mechanisms of these miRNAs in the future. Second, the TCGA database lacks some important postoperative variables (chemotherapy, radiotherapy, hormone therapy), thus we could not carry out a comprehensive analysis and identify the low-risk patients to tailor adjuvant chemotherapy. Third, multicenter, large-scale, prospective studies should be performed to validate this predictive tool before application in routine clinical practice. Fourth, the risk score could accurately discriminate patients with N1, N2 and N3 status. However, the risk score did not accurately discriminate patients with N0 status. Indeed, 21-gene expression Oncotype DX was considered as an accurate molecular tool to discriminate high-risk patients with N0 status and put the indication of adjuvant chemotherapy [28]. Thus, the risk score and 21-gene expression Oncotype DX could be used to identify the low-risk patients whether benefit from adjuvant chemotherapy, regardless of N status.

In conclusion, the current study showed a novel, robust six-miRNA-based prognostic model incorporating six-miRNA signature and clinical risk factors to predict 5-year OS in BC patients. The six-miRNA-based nomogram had higher prognostic value than the conventional TNM stage in BC patients. Furthermore, the six-miRNA signature can effectively identify the low risk patients from the high risk group in BC patients. Therefore, this practical tool has the potential to facilitate individualized treatment decisions for BC patients.

## MATERIAL AND METHODS

### Patients and study design

In this study, the raw counts of BC dataset (Level 3 miRNA expression profiles), including 1044 BC samples and 102 adjacent normal breast tissues were acquired from TCGA data portal in September 2, 2018. The inclusion criteria were included: (1) histologically confirmed invasive BC; (2) both miRNA expression profile and complete survival information available; (3) OS time was more than 1 month. Finally, a total of 984 BC patients with the corresponding clinical features including, age, T stage, N stage, TNM stage, estrogen receptor (ER) status, progestrone receptor (PR) status, human epithelial growth factor receptor 2 (HER2) status were enrolled as primary cohort in this study. And we acquired data from 984 patients randomly assigned 492 patients as the validation cohort based on a computer-generated allocation sequence. Because the application of data abided by the TCGA publication guidelines, the approval of institutional ethics committees was not required.

### Identification of potential OS-related miRNAs of BC patients

The miRNA expression profiles were normalized via the R/Bioconductor package of edger [29]. We defined a miRNA with FDR <0.05 and |log2FC|≥2 of expression level between the 1044 BC samples and 102 adjacent normal breast tissue as DEMs. Firstly, the univariate CPHR analysis was executed to screen for each DEMs associated with OS. Subsquently, these DEMs with a *P*<0.05 were selected into multivariate CPHR analysis to identify the independent prognostic miRNAs of OS (*P* <0.05).

### Construction of risk score formula and miRNA-based prognostic model

Prognostic miRNAs which were distinctly associated with OS in the multivariate CPHR analysis (*P*<0.05) were pointed out to develop the risk score formula. The formula was carried to compute the prognostic risk score for each patient. Using the coefficients obtained from the multivariable CPHR analysis, a risk score formula was built as following: Risk score (miRNA-based classifier) = sum of coefficients × expression level of miRNAs. Moreover, the BC patients were stratified into the high-risk group and the low-risk group via the median risk score as the cutoff value. To provide the oncologists and patients with a quantitative method to achieve individualized survival prediction, we constructed a prognostic nomogram that incorporated both the miRNA-based signature and clinical risk factors using Cox regression model.

### Evaluation of risk score formula and miRNA-based prognostic model

To further assess the predictive performance of the miRNA-based classifier and prognostic model, we measured the area under the curve (AUC) based on time-dependent receiver operating characteristic (ROC) analysis [30]. Furthermore, stratified analysis was conducted to test whether the miRNA-based classifier was associated with OS independent of stages. In addition, calibration curve was used to evaluate the agreement between model predicted outcome and actual outcome. The predictive accuracy of miRNA-based classifier and prognostic model were compared with other risk factors using ROC analysis.

### Target gene prediction and functional enrichment analysis

Potential target genes of prognostic miRNAs were predicted via three online databases, including TargetScan, miRTarBase and miRDB [31–33]. Thus, we confirmed the overlapping miRNA target genes from the three online databases to perform enrichment analysis. The Database for Annotation, Visualization, and Integrated Discovery 6.8 Bioinformatics Tool (DAVID 6.8) was carried out to Gene Ontology (GO) analysis and Kyoto Encyclopedia of Genes and Genomes (KEGG) pathway enrichment analysis.

### Statistical analysis

The Mann-Whitney U test and the χ^2^ test were implemented to compare the associations of continuous and categorical variables between the primary cohort and validation cohort, respectively. Univariate and multivariate CPHR analyses were executed to screen the independent prognostic variables of OS (*P* <0.05). Then, we used the Cox regression coefficients to establish a risk score formula and miRNA-based nomogram. For survival analyses, Kaplan-Meier method was carried out to plot survival curves, which were compared using log-rank tests. The predictive accuracy of each variable was tested via time-dependent ROC analysis. Time-dependent ROC curve analysis is extensively applied in biomedical reports for assessing the predictive accuracy of the six-miRNA signature. It is a graphical display which plots sensitivity estimates (probability of a true positive) against one minus specificity (probability of a false positive) of the six-miRNA signature for all possible threshold values. In a time-dependent ROC analysis, the sensitivity and specificity are determined at each time point to guide important medical decisions [34]. A volcano plot and heat map were drawn using the “ggplot2” package of R software. The primary end point was OS, which was computed the interval from surgery to the date of death from any cause. A value of *P*<0.05 was determined statistically significant. All statistical analyses were conducted with Stata/MP, version 14.0 (StataCorp LP, College Station, TX) and R version 3.4.4 were applied to the statistical analyses.
