# Correction for: A novel six-microRNA-based model to improve prognosis prediction of breast cancer

**DOI:** 10.18632/aging.205643

**Published:** 2024-02-29

**Authors:** Jianguo Lai, Hongli Wang, Zihao Pan, Fengxi Su

**Affiliations:** 1 Guangdong Provincial Key Laboratory of Malignant Tumor Epigenetics and Gene Regulation, Sun Yat-Sen Memorial Hospital, Sun Yat-Sen University, Guangzhou, China; 2 Breast Tumor Center, Sun Yat-Sen Memorial Hospital, Sun Yat-Sen University, Guangzhou, China; 3 Department of Thoracic Surgery, Sun Yat-Sen Memorial Hospital, Sun Yat-Sen University, Guangzhou, China

**Keywords:** breast cancer, microRNA, nomogram, survival, model

**This article has been corrected:** The authors corrected two mistakes in **Table 2**. hsa-miR-147b, which had an expression coefficient of -0.054, was mistakenly labeled as “Up-regulated” but should have been labeled "Down-regulated." hsa-miR-6715a, which had an expression coefficient of 0.072, was mistakenly labeled as "Down-regulated" but should have been labeled "Up-regulated." These corrections do not influence the results or the conclusions drawn in the article.

The corrected version of **Table 2** is provided below.

**Table 2:** Six prognostic miRNAs significantly associated with OS in the primary cohort.

**Name**	**Coefficient**	**Type**	**Down/up-regulated**	**HR**	**95%CI**	**P value**
**hsa-miR-147b**	-0.054	Protective	Down	0.947	0.902-0.995	0.032
**hsa-miR-549a**	0.289	Risky	Up	1.336	1.140-1.564	<0.001
**hsa-miR-6715a**	0.072	Risky	Up	1.075	1.029-1.122	0.001
**hsa-miR-4501**	0.026	Risky	Up	1.026	1.009-1.044	0.004
**hsa-miR-7974**	0.158	Risky	Up	1.171	1.083-1.266	<0.001
**hsa-miR-4675**	0.068	Risky	Up	1.07	1.012-1.132	0.018

Abbreviations: OS, overall survival; CI, confidence interval.

